# Supplementary material for: Interaction of Tryptophan- and Arginine-Rich Antimicrobial Peptide with E. coli Outer Membrane—A Molecular Simulation Approach
Source: Int J Mol Sci. 2023 Jan 19;24(3):2005. doi: 10.3390/ijms24032005 (PMC9916935; doi:10.3390/ijms24032005)
Supplement: Supplementary file 1 [file ijms-24-02005-s001.zip › ijms-2116203-supplementary.pdf]

Supplementary information

Interaction of tryptophan- and arginine-rich antimicrobial peptide with E. coli outer membrane - a molecular simulation approach

George Necula [1], Mihaela Bacalum [2], Mihai Radu [2,*]

[1] Horia Hulubei National Institute for R&D in Physics and Nuclear Engineering, Department of Computational Physics and Information Technologies;

[2] Horia Hulubei National Institute for R&D in Physics and Nuclear Engineering, Department of Life and Environmental Physics

[*] Correspondence: mradua@nipne.ro.

Coarse-Grained MD simulation

The atomistic molecular system consisting of S-LPS *E. coli* membrane model built with five repeating O21 O-antigen serogroup units in conjunction with 12 P6 peptides was converted to Coarse Grained (CG) molecular system using martini22p force field [1]. The atomistic system was converted to CG with the help of the latest version of the Python script martinize.py. The phospholipids PPPE, PVPG and PVCL2 used for the atomistic systems were replaced with DOPC in the CG system. Non-bonded interactions were attenuated at a distance of 12 Å. Energy minimization was achieved with the steepest descent algorithm for 40 ps. The system was heated to 300 K and the pressure was raised to 1 bar. The CG system was equilibrated with molecular dynamics simulation for 3.6 ns with a time step of 20 fs. The temperature was controlled by the Bussi-Donadio-Parrinello thermostat [2] with a coupling interval of 1 ps, while pressure was controlled by the Berendsen barostat [3] semi-isotropic coupling at a compressibility level of 3×10^{-4} bar^{-1} and a coupling time of 1 ps. The MD production simulations were performed with a time step of 20 fs, with the same parameters, with the exception of the Berendsen semi-isotropic coupling method, which was replaced with Parrinello-Rahman barostat [4]. The CG simulation was performed using Gromacs version 2020.3 [5] for a total time of 145 μs.

Structural analysis of OmpF inserted in *E. coli* OM membrane models

There was some variation in the RMSD for all atoms of OmpF between the two types of molecular systems, reaching a plateau of < 3 Å with little drift for the R-LPS *E. coli* membrane model systems, while the S-LPS *E. coli* membrane model systems were more diverse, reaching the plateau between 2.5 - 3.3 Å with some drift. As expected, RMSD values of the β-barrel were similar across all simulations with an average RMSD of ~2.2 Å (Figure S1a). In general, the flexibility of loops L5, L6, L7, and L8 was similar in all the MD simulations, quickly reaching a plateau of ~2.2 Å. The RMSD fluctuations of loop 4 (L4) atoms in all three simulations in conjunction with R-LPS *E. coli* membrane model can be explained by a proportional increase of the number of hydrogen bonds formed between the atoms of this loop with the much more dynamic layer of lipid A (RMSD ~8 Å). The hydrogen bond analysis mirrors the RMSD fluctuations of loop L4, with the plateau values corresponding with 5, 8, and 11 h-bonds/ns for the R-LPS *E. coli* membrane model in interaction with P6, P6m and Pxm peptides, respectively. Because the constriction loop (L3) is located inside of the β-barrel of OmpF channel, it is isolated from external forces, but it does interact with some of the 16 β-strands of the barrel. Intra-protein hydrogen bond analysis revealed an inverse relation between RMSD fluctuations and the level of interaction with the β-barrel interior atoms i.e. lower levels of hydrogen bonds formed with loop L3 were associated with a greater degree of structural flexibility. The progressive reduction from average

> 20 to < 10 h-bonds/ns associated with the gradual increase of RMSD was observed for the OmpF (L3) channel inserted in the S-LPS *E. coli* membrane model in interaction with Pxm peptide (Figure S1b).

Table S1. Molecular systems details

System	S-LPS, 1 O21, P6	S-LPS, 1 O21, P6m	S-LPS, 5 O21, P6m	S-LPS, 1 O21, Pxm	R-LPS, 1 O21, P6	R-LPS, 1 O21, P6m	R-LPS, 1 O21, Pxm	S-LPS, 1 O21, P6	S-LPS, 1 O21
**Protein**	OmpF E.coli	OmpF E.coli	OmpF E.coli	OmpF E.coli	OmpF E.coli	OmpF E.coli	OmpF E.coli	-	-
**No. AMPs**	12	12	12	12	12	12	12	12	-
**No. Atoms**	141448	185853	287360	156563	127728	105008	122081	111443	80245
**No. Water**	32614	47226	75463	37726	29344	21687	27465	24795	15257
**Box size (Å)**	89.3 x 89.3 x 190.3	89.3 x 89.3 x 242.7	89.3 x 89.3 x 379.1	89.3 x 89.3 x 210.5	89.3 x 89.3 x 176.5	89.3 x 89.3 x 147	89.3 x 89.3 x 166.5	81.4 x 81.4 x 191.9	81.4 x 81.4 x 139.9
**Simulation length (ns)**	1000	1000	450	1000	1000	1000	1000	500	1000

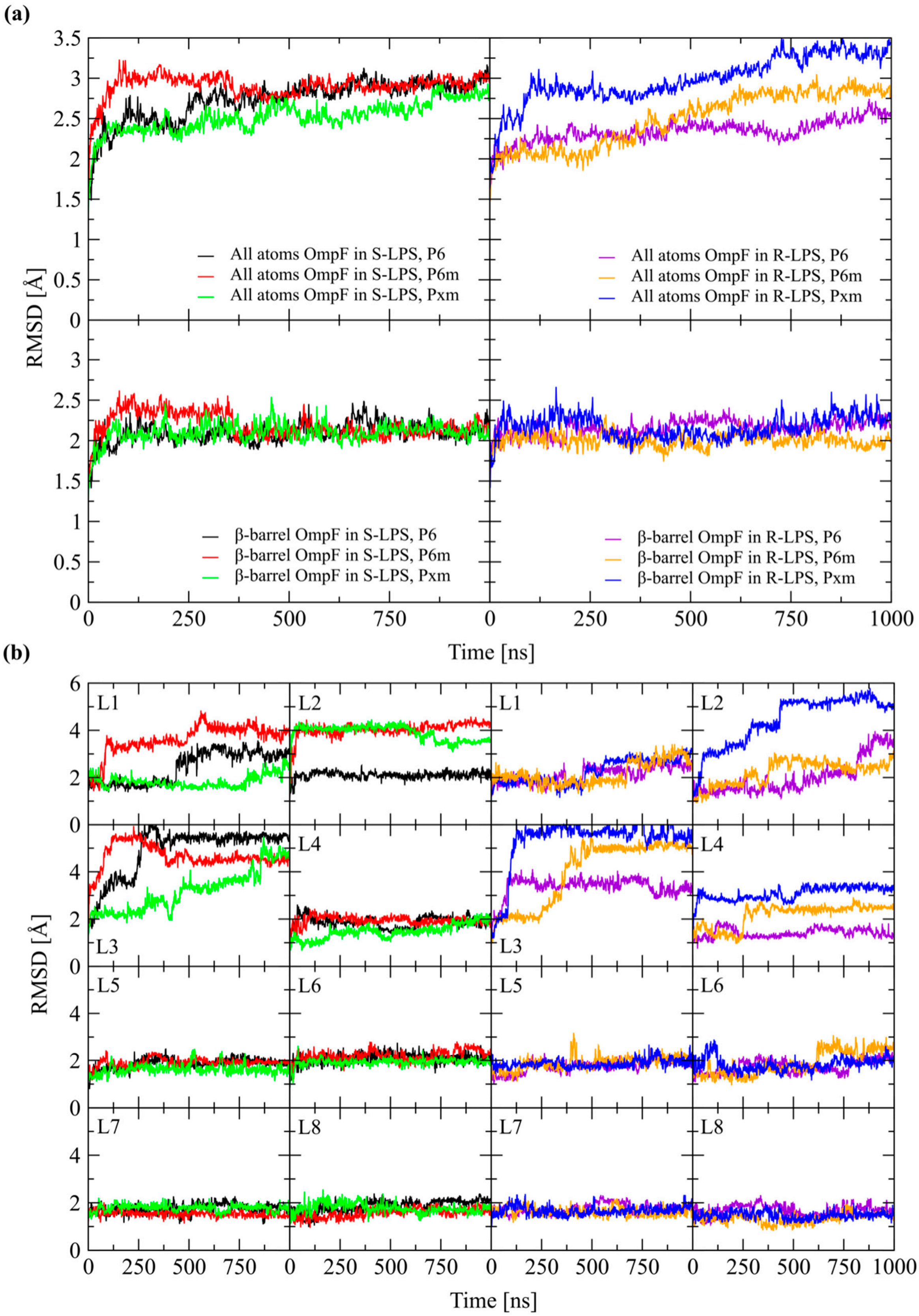

Figure S1. RMSD analysis of OmpF monomer during MD simulation in S-LPS and R-LPS *E. coli* membrane models: **(a)** all atoms and only β-barrel atoms; **(b)** atoms of key OmpF loops.

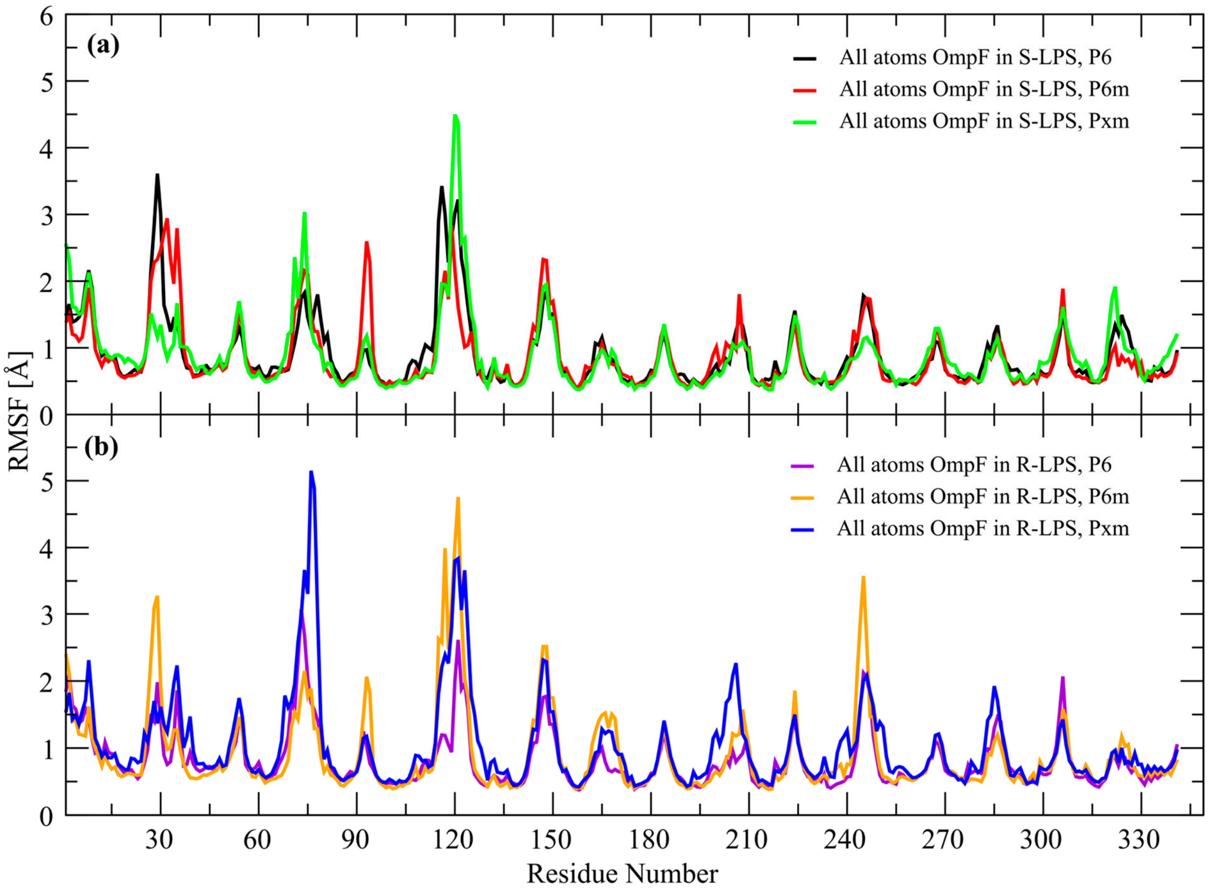

Figure S2. RMSF of OmpF monomer inserted in **(a)** S-LPS and **(b)** R-LPS *E. coli* membrane models.

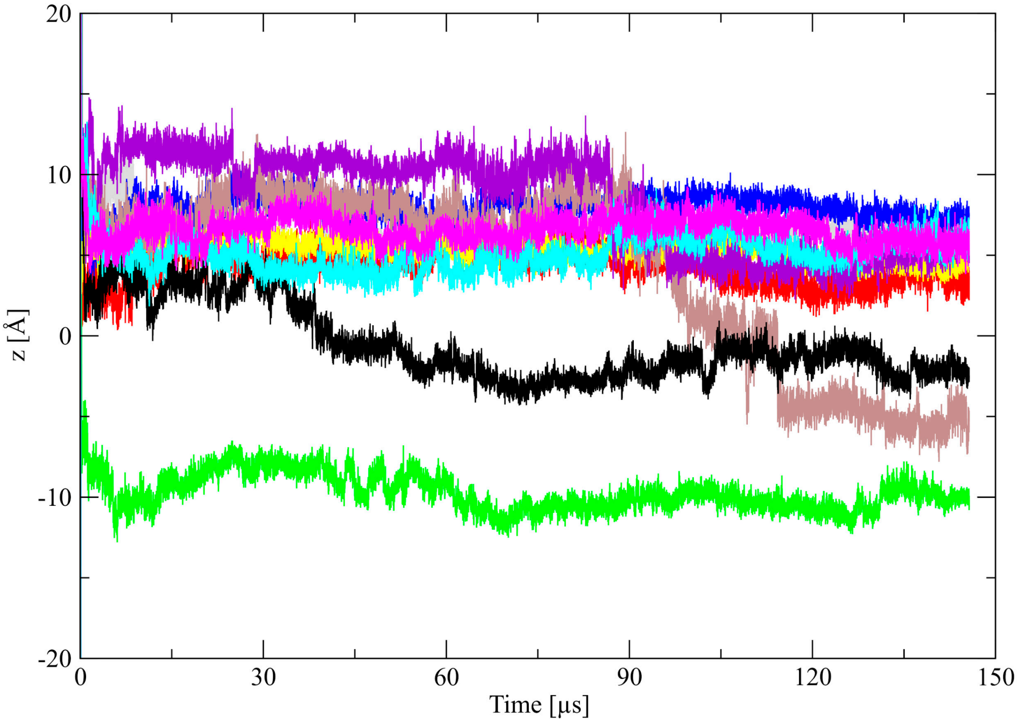

Figure S3. Position of 12 P6 peptide molecules on the z coordinate relative to the S-LPS layer surface as a function of time in a CG-MD simulation.

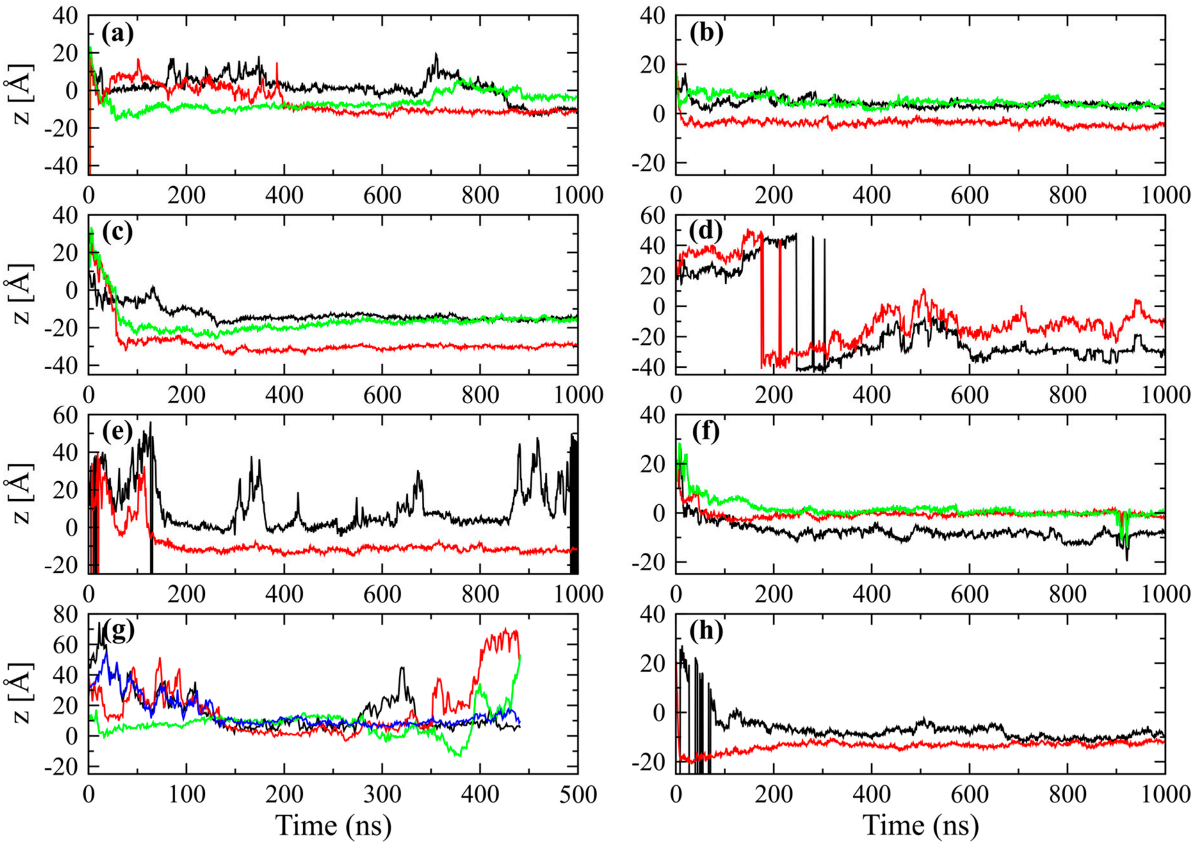

Figure S4. Notable insertions of AMPs in S-LPS (1 O21) *E. coli* membrane model: **(a)** P6, **(c)** P6m, **(e)** Pxm, **(g)** P6m in S-LPS (5 O21), and **(h)** P6 in a system without OmpF monomer; AMPs insertion in R-LPS *E. coli* membrane model: **(b)** P6, **(d)** P6m, **(f)** Pxm.

References

[1] Yesylevskyy SO, Schäfer LV, Sengupta D, Marrink SJ. Polarizable Water Model for the Coarse-Grained MARTINI Force Field. PLoS Comput Biol 2010;6:e1000810. https://doi.org/10.1371/journal.pcbi.1000810.

[2] Bussi G, Donadio D, Parrinello M. Canonical sampling through velocity rescaling. J Chem Phys 2007;126:014101. https://doi.org/10.1063/1.2408420.

[3] Berendsen HJC, Postma JPM, van Gunsteren WF, DiNola A, Haak JR. Molecular dynamics with coupling to an external bath. Journal of Chemical Physics 1984;81:3684–90. https://doi.org/10.1063/1.448118.

[4] Parrinello M, Rahman A. Polymorphic transitions in single crystals: A new molecular dynamics method. Journal of Applied Physics 1981;52:7182–90. https://doi.org/10.1063/1.328693.

[5] Abraham MJ, Murtola T, Schulz R, Páll S, Smith JC, Hess B, et al. GROMACS: High performance molecular simulations through multi-level parallelism from laptops to supercomputers. SoftwareX 2015;1–2:19–25. https://doi.org/10.1016/j.softx.2015.06.001.
